# Ureterocalicostomy for complex upper ureteral stricture: a narrative review of the current literature

**DOI:** 10.1007/s11255-023-03911-8

**Published:** 2024-01-22

**Authors:** Bin Xie, Xuefeng Wang, Xin Zeng, Lingyu Xie, Zhicheng Zeng, Hui Xu

**Affiliations:** 1 First Clinical Medical College, The Gannan Medical University, Ganzhou, China; 2 Urology Laboratory, the First Affiliated Hospital of Gannan Medical University, Ganzhou, China; 3 Department of Urology Surgery, The First Affiliated Hospital of Gannan Medical University, Ganzhou, China

**Keywords:** Ureterocalicostomy, Upper ureteral stricture, Ureteropelvic junction obstruction, Ureteral reconstruction

## Abstract

Upper ureteral stricture is a relatively rare but increasingly encountered condition in clinical practice. While simple stricture can often be addressed through endoluminal treatment or surgical reconstruction, complex upper ureteral stricture poses challenges, particularly in patients with ureteropelvic junction obstruction (UPJO) or perirenal pelvic fibrosis and scarring resulting from previous surgeries. These cases present difficulties for traditional endoluminal and ureteral reconstruction treatments, posing a significant problem for many clinical surgeons. Our study involved a thorough search and comprehensive analysis of the existing literature on Ureterocalicostomy (UC). The literature indicates that UC is a safe and effective treatment for ureteral stenosis. By resecting the renal lower pole parenchyma, it is possible to achieve mucosal anastomosis between the calyceal and ureteral mucosa, leading to the restoration of normal urinary excretion. This technique has emerged as an alternative for treating complex upper ureteral strictures. However, there is a lack of direct comparative studies between open surgery and minimally invasive surgery. Our findings revealed a scarcity of relevant review documents, with most being case reports or retrospective studies conducted in single centers with small sample sizes. Therefore, it is crucial to conduct large-scale, multicenter prospective studies and long-term follow-up to validate the long-term efficacy of UC. This article reviews the development history of UC and focuses on a comprehensive discussion of its indications, surgical techniques, and complications.

Ureteral stricture (US) is a disease characterized by a narrower than normal lumen of the ureter due to various reasons. This condition can lead to varying degrees of upper urinary tract obstruction and hydronephrosis, ultimately impacting renal function [1]. The primary cause of stricture formation is iatrogenic injury (80%) [2]. Other causes include congenital ureteral strictures at the ureteropelvic junction, ureterolithiasis, radiation therapy, infection, retroperitoneal fibrosis, and endometriosis [3–5]. Ureteral stricture can be classified based on its location into lower, middle, and upper ureteral stricture. The lower segment is the most frequently affected area, whereas upper ureteral stricture is more complex compared to the other segments. There are currently various methods available for treating patients with ureteral stricture, including endoscopic treatment, ureteral reconstruction, and kidney autotransplantation (KAT). The choice of treatment depends on several factors: ①The location of the ureteral stricture (upper, middle, or lower ureter). ② The length and severity of the ureteral stricture. ③ The patient’s comorbidities. ④ Any previous radiotherapy or surgery in the planned surgical area. ⑤ The patient’s life expectancy.

Ureteral reconstruction is considered the most effective treatment for ureteral stricture. The surgical techniques commonly used to address upper ureteral stricture include pyeloplasty, ureteroureterostomy (UU), oral mucosal ureteroplasty, and flap pyeloplasty. However, urologists face difficulties in managing complex UPJO and long-segment upper ureteral strictures, as these conditions pose significant challenges. In some cases, segmental disease of the ureter and scar tissue may need to be removed during surgery due to adhesions from previous surgeries and the anatomical characteristics of the renal pelvis and periureter. However, this can be challenging and may result in surgical failure. As a result, many patients end up needing nephrectomy. Traditionally, ileal ureter or KAT are common methods for treating these diseases. However, both procedures are complex, time-consuming, and carry the risk of serious complications [6, 7].

UC is a procedure that is not commonly performed and has specific indications. It offers a treatment option for cases that are clinically complex, which can be beneficial in reducing the need for ileal ureter or KAT. First introduced by Neuwirt in 1947 and initially used for patients with ureteropelvic junction stricture in solitary kidneys, this surgical procedure entails removing the thinned lower pole parenchyma of hydronephrosis and connecting the free ureter directly to the inferior renal calyx to ensure efficient urinary drainage [8]. Subsequently, numerous medical institutions have documented their experiences with UC, providing evidence of its safety, effectiveness, and favorable outcomes [9–13]. Due to the complexity of UC, open methods have been traditionally used in the past. However, with the continuous exploration of surgeons and the advancements in laparoscopic technology, laparoscopic or robot-assisted laparoscopic ureterocalicostomy (LUC or RALUC) has been increasingly performed on patients with severe hydronephrosis caused by complex upper ureteral stricture and obstruction. These procedures have shown satisfactory results [14–16]. A comprehensive search was performed using the PubMed database by entering the search term “ureterocalicostomy” to collect relevant studies on UC. The key studies are summarized in Table 1.

**Table 1:** The main studies of ureterocalicostomy

Authors	Year	Patients	Male:female	Median age	Surgical procedure	Mean operative time (min)	(Mean) blood loss (ml)	Postoperative hospital stay (d)	(Mean) follow-up/months	Success rate (%)
Neuwrit et al. [8]	1947	1^a^	N/A	N/A	UC	N/A	N/A	N/A	NR	100%
Jameson et al. [17]	1957	1	N/A	N/A	UC	N/A	N/A	N/A	9	100%
Hawthorn et al. [18]	1976	3	N/A	N/A	UC	N/A	N/A	N/A	10.7	100%
Selli et al. [11]	1982	10	5:5	39	UC	NR	NR	NR	14.7	90%
Jarowenko et al. [19]^*^	1985	1^b^	1:0	36	UC	N/A	N/A	N/A	5	100%
Guiter et al. [20]^*^	1985	135	N/A	N/A	UC	N/A	N/A	N/A	N/A	> 90%
Mesrobian et al. [21]	1989	21	12:9	9	UC	NR	NR	NR	6–84	90%
Ross et al. [12]	1990	7	6:1	41	UC	NR	NR	NR	30	71%
Cherullo et al. [22]	2003	10^c^	–	–	LUC	165	NR	145	1.6	60%
Gill et al. [23]	2004	2^d^	0:2	28	LUC	231	137.5	2	7.5	100%
Ben et al. [24]	2005	5	N/A	N/A	UC	N/A	N/A	N/A	21	100%
Haouas et al. [13]	2005	17	N/A	N/A	UC	N/A	N/A	N/A	NR	71%
Matlaga et al. [25]	2005	11	7:4	38	UC	292.2	372.5	5.1	10.1	100%
Korets et al. [26]	2007	1^e^	1:0	43	RALUC	300	500	3	2.3	100%
Casale et al. [27]	2008	9	NR	6.5	RALUC	168	NR	1	12	100%
Mijan et al. [28]	2009	1	0:1	35	UC	NR	NR	7	30	100%
Schimpf et al. [29]	2009	1	0:1	32	RALUC	180	NR	2	36	100%
Radford et al. [30]	2011	13	5:8	9.3	UC(*n* = 12) RALUC(*n* = 1)	(170) (151)	NR	5	31.2	92%
Osman et al. [31]	2011	22	14:8	36.3	UC	178	534	7.6	26.7	73%
Arap et al. [32]	2014	6	0:6	20.1	LUC	215	100	4.5	30	100%
Raj et al. [33]	2017	1	1:0	25	UC	NR	NR	NR	6	100%
Nishimura et al. [34]	2017	1	0:1	10	LUC	379	NR	6	29	100%
Srivastava et al. [35]	2017	72	49:23	28.9	UC(*n* = 34) LUC(*n* = 38)	170.5	320.6	7.3	60.3	69.5%
Astolfi et al. [36] ^*^	2018	1	1:0	62	UC	29^**^	NR	NR	6	100%
Shi et al. [37]	2020	12	9:3	49.2	UC (*n* = 9) LUC(*n* = 3)	198.5 111.6	8.9 6.2	148.8 82.9	27	92%
Ramanitharan et al. [14]	2020	6	2:4	33.7	RALUC	178	115	6.1	15	100%
Divjak et al. [38]	2021	1	1:0	3.5	LUC	NR	NR	NR	12	100%
Adamic et al. [39]	2021	4	1:1	8	RALUC	208	27.5	3	53.5	100%
Ansari et al. [40]	2021	25	22:3	7	UC(*n* = 20) LUC(*n* = 4) RALUC(*n* = 1)	NR	NR	6	22	92%
So et al. [15]	2022	1	0:1	28	RALUC	185	NR	NR	1.4	100%
Mittal et al. [41]	2022	24	17:7	5.1	RALUC	NR	NR	NR	16.1	92%
Esposito et al. [42]	2022	15	11:4	10.1	LUC(*n* = 10) RALUC(*n* = 5)	157.6	NR	2.8	37.2	100%
Salehipour et al. [43]^*^	2023	1	0:1	20	UC	N/A	N/A	N/A	1	100%
Nunes et al. [16]	2023	1	0:1	24	LUC	130	NR	2	12	100%

*UC* ureterocalicostomy, *LUC* laparoscopic ureterocalicostomy, *RALUC* robot-assisted laparoscopic ureterocalicostomy, *NR* not reported

^a^The first report about UC; ^b^the first report about UC in kidney transplantation; ^c^the first report about LUC in animals; ^d^the first report about LUC in human; ^e^the first report about RALUC in human

*Reports about UC in kidney transplantation

**Anastomosis time

## Open ureterocalicostomy

In 1947, Neuwirt conducted the first UC on a patient who had a solitary kidney. In this procedure, he pulled the everted ureter into the inferior renal calyx through the cortex and then directly sutured the ureter and the renal capsule [8]. Jameson et al. [17] reported a case of severe hydronephrosis in a child with a solitary kidney caused by intrarenal pelvic stricture and obstruction. After unsuccessful attempts at intraluminal treatment, Jameson and colleagues decided to perform UC. During the operation, the part of parenchyma of the lower pole of the kidney was excised, and then, the ureter was pulled into the kidney and anastomosed with the calyceal mucosa. Finally, the ureter was sutured to the renal parenchyma. On the 22nd day post-operation, the nephrostomy tube was removed, and the urography showed no significant changes compared to pre-operation. Subsequently, the patient was readmitted to the hospital due to low-grade fever and pyuria. Considering the possibility of anastomosis stenosis, a second operation was decided upon. During the operation, severe scarring, sclerosis, and contracture were observed at the lower end of the kidney, which compressed the ureteral part of the lower pole parenchyma. After resecting the fibrotic and shrunken lower end of the kidney, re-anastomosis was performed. The nephrostomy tube was removed 9 months after the operation. The patient remained asymptomatic and showed no abnormalities in urine tests.

Later, inspired by Jameson's second ureterocalicostomy technique, Hawthorne et al. [18] proposed and refined the surgical procedure. They emphasized the following principles for performing the operation: (1) Sufficient resection of the lower renal pole cortex should be done up to the anastomosis to prevent compression of the ureter caused by postoperative contractile fibrosis of the renal cortex. (2) A longitudinal incision should be made and the ureter should be unfolded on one side to ensure maximum patency of the anastomosis. Non-circumferential sutures should be used for the anastomosis. (3) A retrograde ureteral catheter was inserted during cystoscopy to assist in locating the ureters. The ureteral stent, which was of sufficient size, was replaced and anastomosis was performed. Additionally, nephrostomy drainage was also implemented. In their report, UC was successfully performed in 3 complicated cases involving children. Angiography results at the 18th, 6th, and 8th months of postoperative follow-up showed smooth drainage of the anastomosis and significant improvement or disappearance of hydronephrosis. The children did not experience any discomfort symptoms.

Subsequently, many scholars reported the application of this technique in patients with ureteral stricture. In a study by Ben et al. [24], UC was performed in 5 patients with congenital stricture at the ureteropelvic junction, and lower pole nephrectomy was carried out in all cases. After an average follow-up period of 21 months (ranging from 20 to 27 months), satisfactory postoperative radiological results were observed in 3 patients. One patient experienced rapid stenosis but was successfully treated endoscopically, while another patient had a poor outcome and ultimately required nephrectomy. As reported by Mijan et al. [28], UC has been found to be a viable treatment option for intrapelvic UPJO resulting from changes in the fusion, rotation, or location of the kidney. It can also be used for severe peripelvic fibrosis cases where previous pyeloplasty or renal surgery has been unsuccessful. According to Radford et al. [30], UC is a reliable method for resolving severe pelvic ureteral junction obstruction caused by horseshoe kidney, recurrent pelvic ureteral junction obstruction, and anatomical abnormalities. In their study, they performed UC on 13 children at the same hospital. Out of these cases, 12 (92%) showed good functional results with an average postoperative follow-up time of 2.6 years (ranging from 0.3 to 7.0 years), as assessed by ultrasound and/or isotope imaging. The imaging revealed reduced ureteral dilatation and improved drainage. Kalathia et al. [44] performed UC for a patient with severe hydronephrosis caused by pelvic ectopic kidney with UPJO. The patient's unique anatomical condition presented a challenge as the renal pelvis was facing forward and the ureter passed backward. To overcome this, the researchers performed a mucosa-to-mucosa anastomosis between the ureter and the middle calyx of the kidney. The postoperative results were highly successful.

## Animal experiments

In 2003, Cherullo et al. [22] conducted an animal experiment involving LUC on 10 surviving female pigs. They established a model of upper ureteral stricture by laparoscopic ligation and intentional electrocautery of the ureteropelvic junction and proximal ureter, resulting in an average obstruction interval of 6.3 days. This led to periureteral fibrosis, scars, and severe hydronephrosis, mimicking the clinical situation of UPJO. The average length of ureteral stricture was 2.2 cm (range 1.7–3.1 cm). To restore ureteral continuity, the lower kidney was transversely truncated and the free ureteral stump was anastomosed to the open lower calyx. The average operation time was 165 min, with an average blood loss of 145 ml. The stent was left in place for an average of 8.7 days. Complications included 1 case of renal artery injury and 2 cases of small intestinal serosal tear. The laparoscopic repair successfully addressed the serosal injuries. During a mean follow-up period of 47 days (ranging from 25 to 65 days), excretory urography and retrograde ureterography demonstrated satisfactory excretion of contrast media, unimpeded urine drainage, and absence of any obstruction or fistula. Post-mortem examination conducted after euthanizing all pigs did not reveal any signs of urine leakage around the kidneys, and histological analysis indicated satisfactory healing of the urothelium without noticeable fibrosis or scar formation.

The follow-up time of this study was short, but the good postoperative results provided evidence that LUC was a safe and feasible treatment option for upper ureteral stricture.

## Laparoscopic ureterocalicostomy

On the basis of the success of animal experiments, Gill et al. [23] first reported the cases of LUC for the treatment of UPJO in 2004, which was the first time in the world to use LUC in human body. One patient was diagnosed with upper ureteral obstruction and multiple secondary stones were found at the lower end of the renal calyx. The other patient had UPJO due to previous surgical failure, accompanied by renal pelvis scarring and noticeable hydronephrosis. Both surgeries were successfully performed using laparoscopy, with no complications. The operation time for the first patient was 5.2 h (including 1.5 h for renal stone removal) and 2.5 h for the second patient. The blood loss was 200 ml and 75 ml, respectively. Both patients were discharged 2 days after surgery. The double J stents were removed at the 8th and 5th week for the first and second patients, respectively, followed by retrograde ureteropyelography. The results indicated unobstructed calyx ureter anastomosis for both patients. At the 9-month follow-up after the operation, Patient 1 did not report any discomfort, and there was a significant improvement in the imaging examination compared to before the operation. However, Patient 2 still experienced some degree of pain and discomfort at the 6-month follow-up after the surgery. The imaging examination revealed poor renal function on the same side (25%), leading to a nephrectomy being performed at a local hospital. These short-term follow-up results confirmed the technical feasibility of LUC. However, long-term follow-up results and large-sample clinical studies are still needed to evaluate the effectiveness and persistence of the procedure.

In a small sample of study conducted by Arap et al. [32], the mid-term outcomes of LUC were reported in six patients with complex upper urinary tract obstruction. Over a median follow-up period of 30 months (ranging from 8 to 56 months), all patients showed improvement in their clinical and imaging conditions, with unobstructed anastomosis and disappearance of symptoms. Shi et al. [37] conducted an analysis on the efficacy of UC in treating secondary long-segment proximal ureteral stricture. Out of the 13 patients who underwent the operation, 12 were followed up for an average of 27 months. The results indicated that the operation successfully relieved hydronephrosis, with no occurrence of anastomotic stricture. Additionally, there were significant decreases in serum creatinine and cystatin C levels, while the glomerular filtration rate showed a significant improvement compared to pre-operation values. During the perioperative period, 3 patients experienced fever, and 2 patients had symptoms of hematuria and lower urinary tract irritation. However, these symptoms improved after receiving appropriate symptomatic treatment.

In the case of hydronephrosis caused by renal malrotation in children, LUC has also been reported. After a follow-up of 1 month, the dilatation of the renal pelvis had completely subsided. The ureteral stent was removed after 3 months. A radionuclide scan conducted 1 year after the surgery indicated that the bilateral renal function levels were comparable, ultrasound showed no remaining hydronephrosis, and the urinary flow of the affected kidney was unobstructed [38]. In two case reports [16, 34], both patients underwent UC due to obstruction of the ureteropelvic junction following pyeloplasty. The operation was successfully performed, and postoperative follow-up results demonstrated significant improvement in hydronephrosis with no evidence of anastomotic obstruction. There are limited case series on LUC. However, the high success rate of the surgery demonstrates that it is a safe and effective option for patients with complex upper urinary tract obstruction.

## Robot-assisted laparoscopic ureterocalicostomy

In 2007, Korets et al. [26] reported the first case of da Vinci robot-assisted laparoscopic ureterocalicostomy. The procedure was performed on a patient who had refractory upper ureteral stricture as a result of multiple stone interventions. A 43-year-old man underwent three extracorporeal shock wave lithotripsy (ESWL) procedures and two left percutaneous nephrolithotripsy (PCNL) procedures at different institutions to treat a large left kidney stone. Postoperative imaging examination revealed narrowing of the left upper ureter and only 25% left renal function. The patient initially received endoscopic treatment for the upper ureteral stricture, followed by regular replacement of the ureteral stent. However, the left renal function did not improve. Subsequent CT scan results indicated atrophy of the lower pole of the kidney and the presence of residual stones in some calices. After a comprehensive discussion of the treatment options, the author decided to perform RALUC for the patient. The surgery was successful with no complications during the perioperative period. The operation took 300 min and there was a blood loss of approximately 500 ml. The patient was discharged on the 3rd day after the surgery. The ureteral stent was removed at the 4th week, and intravenous urography was conducted 10 weeks after the operation. The results indicated smooth urine excretion without any signs of obstruction. This was the first RALUC reported in the world. The short-term follow-up revealed no complications or adverse outcomes, indicating a successful surgery. The authors concluded that RALUC can be considered as an alternative to endoscopic management for ineffective proximal ureteral strictures. However, long-term follow-up results for this particular case were not reported.

Due to the favorable outcomes observed in the treatment of upper ureteral stricture using robot-assisted laparoscopy technology, several centers subsequently reported the utilization of RALUC for managing intricate cases of secondary ureteropelvic junction stricture. In a long-term follow-up report [29], a woman underwent RALUC to treat ureteropelvic obstruction. The results remained favorable after 3 years of follow-up. About 2 years post-surgery, the patient experienced abdominal pain and worsening hydronephrosis at 3 months of gestation, leading to the performance of a percutaneous nephrostomy. Postpartum nephrography revealed that the anastomotic stoma was unobstructed, and subsequently, the nephrostomy tube was removed.

A multicenter pediatric cohort study [41] reported that RALUC was a safe and effective treatment option for UPJO after failed pyeloplasty or due to complex anatomy. The study included 24 patients with secondary UPJO or extensive intrarenal scarring of the renal pelvis, 21 of whom had a history of pyeloplasty. No Clavien–Dindo grade III–V complications were observed in any of the patients within one month after surgery. During a median follow-up of 16.1 months, symptoms and hydronephrosis improved in 22 (92%) patients without requiring further intervention. The surgical success rate was 92%. However, two patients (8%) underwent postoperative endoscopic interventions, which were unsuccessful, ultimately leading to nephrectomy.

## Ureterocalicostomy in kidney transplantation

The treatment of choice for end-stage renal disease (ESRD) is kidney transplantation. As the number of kidney transplants continues to increase, the incidence and complexity of procedure-related complications also increase [36, 45]. Ureteral stricture is one of the most common long-term urinary complications, occurring in 3–8% of transplant patients. The severity of ureteral stricture depends mainly on its location and length [46, 47]. Studies have shown that UC is an effective method to avoid kidney resection in patients with secondary ureteral stricture after kidney transplantation [19, 36, 43].

Jarowenko et al. [19] reported the initial instance of UC for ureteral obstruction resulting from ischemia following kidney transplantation. The ureteral stent and nephrostomy were removed 6 weeks post-surgery. Subsequent excretory urography after 5 months of follow-up indicated unobstructed passage of the contrast agent, and the serum creatinine level remained stable at 2.0 mg/dl.

Salehipour et al. [43] reported a case of ureteral obstruction after renal allograft transplantation, in which UC combined with lower pole nephrectomy was performed. The patient experienced oliguria 2 months after the transplantation. Ultrasonography of the abdomen and pelvis revealed severe hydrops in the transplanted kidney, which was relieved after percutaneous nephrostomy. Subsequent pyelography showed severe dilation of the renal pelvis and a small intrarenal pelvis. Endoscopic treatment was not feasible due to the obstruction preventing the passage of the guide wire. Ultimately, an open UC combined with lower pole nephrectomy was chosen. The operation was successful, resulting in normal postoperative urine output and a return of creatinine levels to normal. At the 1-month follow-up, ultrasonography demonstrated significant improvement in hydronephrosis, and creatinine levels remained within the normal reference range. The short-term follow-up results are satisfactory, and we look forward to receive the authors' report on the medium-term and long-term follow-up results.

## Discussion

UC has become a feasible technology through the technical improvement of many scholars. As reported by many scholars, we also have obtained favorable outcomes by performing UC in patients with intrarenal renal pelvis and previous surgery. We recognize that traditional methods such as ureterectomy and anastomosis, as well as oral mucosal ureteroplasty, pose challenges in such cases and may result in damage to the renal pedicle during the operation. Endoscopic surgical treatment is only suitable for short ureteropelvic junction stenosis and has limited long-term effectiveness. The surgical procedure involving ileal replacement ureter or autologous kidney transplantation is highly intricated and carries a high risk of postoperative complications. In contrast, the UC avoids the procedure of separating the renal hilum vessels. For most patients with severe hydronephrosis caused by pelvic ureteral obstruction, the process of UC is relatively straightforward and less bleeding.

Most scholars believe that the indications for UC are as follows [11, 30, 37, 41, 43, 44, 48]: (1) congenital ureteropelvic junction stenosis; (2) UPJO secondary to complex anatomical abnormalities of the kidney, such as horseshoe kidney, renal malrotation, ectopic pelvic kidney, and intrarenal small renal pelvis; (3) obstruction of the ureteropelvic junction after pyeloplasty; (4) recurrent kidney stones with upper ureter stenosis; (5) long upper ureteral avulsion; (6) upper ureteral stricture greater than 4 cm in length; (7) secondary upper ureteral stricture after kidney transplantation; (8) other causes of upper ureteral stricture, such as tuberculosis and tumors. However, it is not advisable to perform this surgery in patients with severe infection, anticipated high tension in the anastomosis of the ureter and lower pole calyx, or poor renal function on the affected side. The maximum length of UC for the treatment of ureteral stricture is currently unknown. In a study [37], the average stenosis length for open and laparoscopic ureterocalicostomy for upper ureteral stricture was found to be 4.3 and 4.7 cm, respectively.

**Fig. 1:**
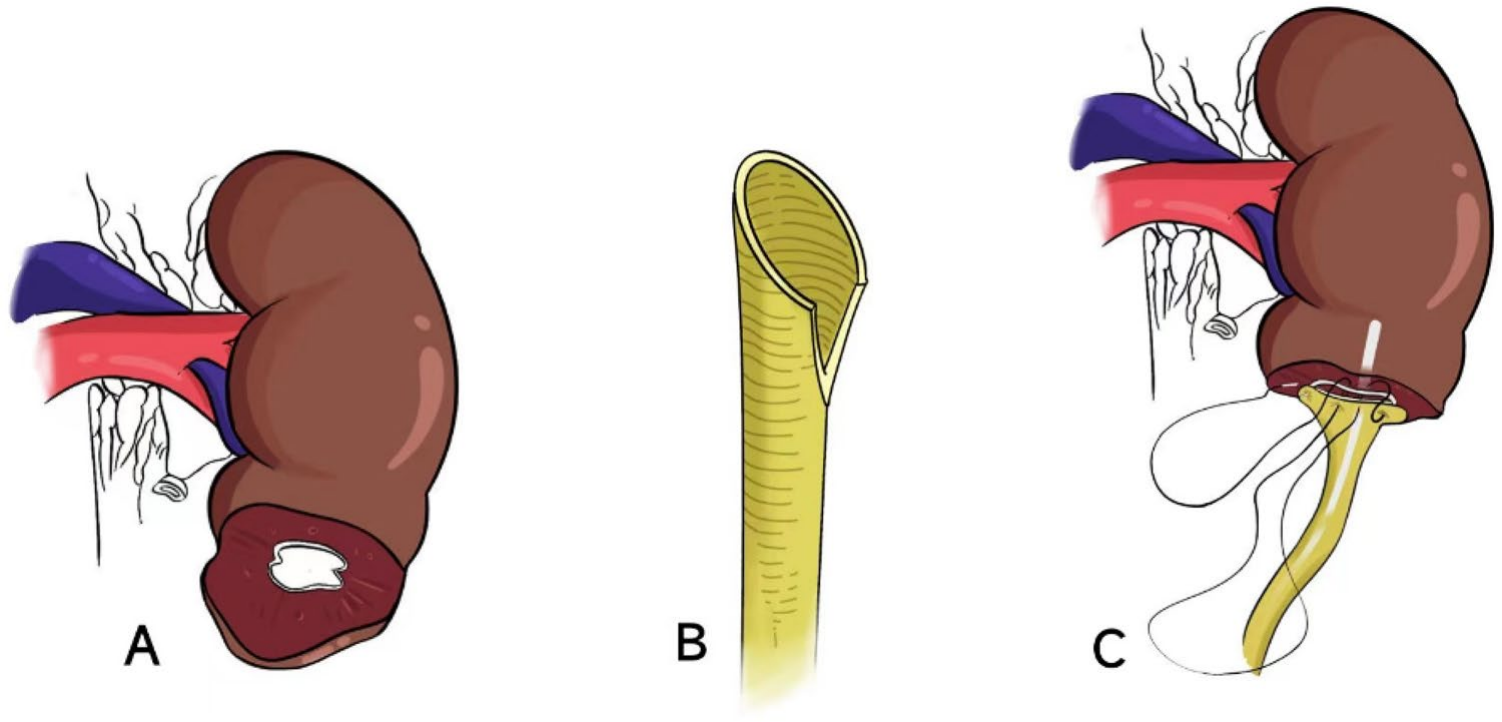
**A** a. Free and ligate the upper ureter; b. lower pole parenchyma excision and expose the outlet of the lower calyx of the kidney. **B** Make an incision on one side of the ureter. **C** a. Place a ureteral stent; b. mucosa-to-mucosa anastomosis of renal calyx and ureter

Regarding surgical technique, it is important to note the following points [18, 39, 42]. First, during UC, it is necessary to make an incision or resect the parenchyma of the lower pole of the kidney in a precise manner (Fig. 1A). This helps in providing better exposure of the surgical area, facilitating reconstruction, and preventing postoperative strictures caused by excessive renal parenchymal fibrosis at the anastomosis site. When Jameson and colleagues performed a UC without resecting enough lower pole cortex, scarring and contracture developed, resulting in obstruction of the segment of the ureter within the kidney [17]. They successfully performed the anastomosis again after resecting the lower pole of the kidney and ureter. Subsequently, several scholars have emphasized the importance of lower pole nephrectomy during UC. Without resecting the lower pole, there is a risk of recurrent ureteral obstruction due to cortical fibrosis covering the intrarenal segment of the ureter [18, 33]. Second, during the operation, it is important to carefully dissect and preserve the blood supply of the ureter to minimize postoperative complications. To ensure a tension-free and optimal mucosa-to-mucosal anastomosis with the calices (Fig. 1C), it is necessary to make an incision on one side of the ureter, which provides sufficient tissue (Fig. 1B). This promotes healing, enhances the return of function, reduces the risk of adverse outcomes, and improves surgical success. Additionally, a ureteral stent should be placed to maintain a smooth urine flow and prevent postoperative stricture or occlusion (Fig. 1C). It is also recommended to insert a drainage tube around the anastomosis.

In the field of urology, laparoscopic surgery has become a routine procedure for treating urinary system diseases. The success of LUC relies on following the fundamental steps of open UC, adhering to established principles of open surgery, and demonstrating proficient hemostasis and cutting capabilities. Compared to traditional open surgery, the laparoscopic technique offers several advantages including reduced invasiveness, faster recovery, and shorter hospital stay. However, with the advancements in laparoscopic technology, robot-assisted laparoscopic technology is increasingly being utilized for ureteral reconstruction. This approach provides even more significant advantages over traditional laparoscopic surgery, such as the use of a robotic platform, 3D visualization, a multi-directional intracarpal system, and improved ergonomic design. These special features greatly facilitate the internal suturing of UC [49]. Previous studies have reported a success rate of > 90% for RALUC [14, 27, 41, 50]. Additionally, the literature suggests that RALUC is the preferred option for patients with recurrent pelvic ureteral junction obstruction [42]. However, there is currently no comparative study available that directly compares LUC and RALUC.

According to a small series of studies comparing open versus laparoscopic ureterocalicostomy [37], the results showed that the laparoscopic technique had lower operative time and blood loss compared to open surgery. However, there was no significant difference in postoperative hospital stay. The authors did not analyze possible reasons for this difference, and they did not compare the advantages and disadvantages of postoperative recovery between the two surgeries in detail. Currently, there have been no direct comparison studies between open surgery and minimally invasive surgery. Therefore, further clinical research is urgently needed to determine the pros and cons of these two types of surgery to provide the best choice for surgeons and patients.

The most common complications of UC include renal parenchymal hemorrhage, infection, urine leakage, and recurrent anastomotic stricture [11].

Effective hemostasis is a crucial concern during resection of the renal lower pole parenchyma [32]. In the past, most reported cases of UC exhibited thin renal parenchyma, particularly in the lower pole of the kidney. These cases also showed relatively minimal bleeding when a portion of the renal parenchyma was removed [32, 34, 51, 52]. In the absence of soft-coagulation hemostatic techniques, surgeons commonly employed suture ligation to control bleeding from the margins. Renal artery clamping was only performed in cases where significant bleeding was anticipated or when the parenchyma of the infrarenal pole was slightly thicker (with very few patients requiring removal of a large amount of infrarenal parenchyma). The question of whether renal arteries should be routinely clamped during UC was initially explored by Ross et al. [12]. In their study, they found no significant decrease in blood loss when the renal arteries were clamped in three patients. As a result, they suggested that clamping the renal arteries should only be considered when local control of hemorrhage is inadequate.

In recent years, hemostatic techniques have developed rapidly. A study [39] demonstrated that the use of a harmonic scalpel can reduce blood loss during this procedure, and none of the patients in their cases required blood transfusions. However, it is essential to exercise caution near the collection system to avoid potential risks, such as anastomotic rupture and urine leakage, as the harmonic scalpel generates significant energy and heat. Yagihashi et al. [53] reported a case of LUC in a patient with symptomatic UPJO. They utilized a minimally invasive sutureless technique using the VIO soft-coagulation system during the resection of the lower renal pole. Hemostasis was successfully achieved on the surface of the lower pole nephrectomy. This technology offers several advantages over traditional coagulation systems [54]: ① It eliminates the production of sparks that can lead to tissue carbonization; ② It can degrade proteins up to a depth of < 2 mm from the resection surface, thereby reducing potential damage to kidney function. Regardless of whether hemostasis is achieved through suture ligation, harmonic scalpel, or VIO soft-coagulation system, our primary objective remains the same: to prevent bleeding and minimize postoperative complications.

Recurrent urinary tract infection is a significant postoperative complication and can occur in 30% of patients [35]. Despite taking antibiotic precautions during surgery, there is still a risk of infection. Infection can lead to symptoms such as fever, pyuria, and abdominal pain. Early diagnosis and prompt treatment of infection are crucial, and appropriate antibiotics are usually required to control the infection.

Urine leakage may occur due to an incomplete anastomotic site or postoperative infection. Vigilant postoperative observation and early identification of urine leakage are essential for its prevention and treatment.

Recurrent anastomotic stricture is a common complication, often caused by ischemic damage to the renal parenchyma or ureter. This damage leads to fibrosis at the anastomotic site or scarring due to urine extravasation, which in turn limits ureteral peristalsis [42]. This is the main reason why the patient had to undergo nephrectomy. To minimize the risk of recurrence, several measures can be taken, including resecting a large amount of renal parenchyma, maximizing exposure of the anastomotic site, performing a tension-free anastomosis, and placing a ureteral stent [27].

Resection of part of the renal parenchyma at the lower pole of the kidney is considered a crucial step in UC, which is widely accepted among scholars in this field. In cases of severe hydronephrosis, only a small amount of thin-walled renal parenchyma is removed from the lower pole of the kidney, resulting in minimal impact on overall renal function. However, the renal function of the affected kidney generally improves after the obstruction is relieved through surgery, which is supported by the existing literature [32, 34]. In cases where the renal parenchyma is thicker, removing a portion of it helps to achieve better anastomosis between the inferior renal calyx and the ureter, and prevents the anastomosis from narrowing again. Although some renal parenchyma is lost, there is still a degree of improvement in renal function after the obstruction is resolved [25, 30]. In the published literature, most studies have reported improved renal function after surgery, and we analyzed the possible reasons for this: ① After undergoing UC, a new drainage channel is established from the inferior renal calyx to the ureter, allowing for complete urine drainage. This relieves hydronephrosis significantly, reduces intrarenal hydrostatic pressure, and enables the release of compressed nephrons, leading to a gradual return to normal function. ② Upper urinary tract obstruction leads to long-term severe hydronephrosis in the kidney, and the thinned renal parenchyma has long lost its normal function due to insufficient blood supply because of vascular insufficiency, and the removal of a part of non-functional renal parenchyma at the lower pole of the kidney has a minimal effect on the renal function of the affected side. Matlaga et al. [25] reported their experience with UC treatment in 11 adult patients. All patients experienced resolution of obstruction, and the mean split isotope renal function (SIRF) after UC improved from 54.6% to 60.1%. Similarly, Osman et al. [31] conducted a study with comparable results. In their study, all patients who underwent calyceal ureteral anastomosis had a portion of the renal lower pole parenchyma removed. The operation was successful in 16 patients (73%). Not only did the postoperative renal function not decrease, but it actually improved. The SIRF increased from an average of 27.77% ± 9.48% before surgery to 33.2% ± 13.82% after surgery. The authors compared these results with the better postoperative renal function reported by Matlaga et al. and suggested that the relatively good renal function before surgery may be the reason for this difference.

Regarding the criteria for successful surgery, clinical symptoms should disappear, hydronephrosis and renal function should improve or stabilize, and the absence of a ureteral stent or nephrostomy tube indicates satisfactory results. It is important to note that a single examination result should not be used as the sole determinant of surgical success or failure. Instead, a comprehensive judgment should be made by considering the clinical symptoms, laboratory tests, and imaging results during postoperative follow-up. According to Ansari et al. [40], ultrasonography or diuretic renogram parameters alone cannot be used as a sole determinant of surgical success or failure. However, they should be combined for postoperative follow-up after UC. Along with imaging examinations, blood levels of creatinine and cystatin C are also indicators of good renal function. Some studies have indicated a positive correlation between cystatin C levels and the extent of renal function damage [55]. On the other hand, serum creatinine levels can be influenced by various factors such as diet and age, and improvement in glomerular filtration rate will only occur when renal function drops below half [56]. Therefore, it is more valuable to utilize serum creatinine and cystatin C to evaluate postoperative renal function during follow-up. The success rate of UC varies among different populations. In the adult literature, it has been reported to have success rates ranging from 69.5% to 100% [31, 35]. In the pediatric literature, the success rates range from 70 to 90%, or even higher [12, 21, 27, 30, 41].

The success rate of UC is generally satisfactory in both adults and children. However, it is important to acknowledge that there are instances of surgical failure in clinical practice. The failure rate can be attributed to various factors, and it is worthwhile to investigate the underlying reasons. Srivastava et al. [35] conducted a study on the long-term outcomes and factors associated with the failure of UC. Their multivariate analysis revealed that poor cortical thickness and low glomerular filtration rate were independent predictors of surgical failure. In a retrospective study conducted in 2020 [57], the authors analyzed the long-term results of UC and concluded that the parenchymal thickness of the inferior renal pole less than 10 mm was the most significant predictor for evaluating the effectiveness of UC. Hence, the thickness of the renal inferior pole parenchyma plays a crucial role in determining the need for UC. It is essential to evaluate the patient's preoperative examination results to make an informed decision and minimize the chances of surgical failure. While both studies have indicated that renal parenchymal thickness impacts the success rate of the procedure, there is no unified and specific numerical standard as a factor to predict postoperative efficacy.

According to a study [20], UC has a success rate of over 90% for patients with secondary upper ureteral stricture after kidney transplantation. Currently, open UC is used to treat severe upper ureteral obstruction after kidney transplantation. This is because the surgery needs to be performed in a more limited transplant site and in the previously operated surgical area. Previous literature [19, 36, 43, 58] had reported good curative effects. To date, there have been no reports on the utilization of robot-assisted laparoscopy in kidney transplantation procedures. The advanced capabilities of robotic technology, such as three-dimensional visualization and enhanced freedom of movement, offer surgeons improved visibility and precise anatomical details [50]. Consequently, there is a potential for this technique to be employed in cases of upper ureteral obstruction following kidney transplantation, offering clinical surgeons a viable solution to address such challenges and potentially minimize patient discomfort during the recovery period.

## Limitations of the current literature

Currently, there are a limited number of studies available on UC. Most of these studies consist of case reports or retrospective studies with small sample sizes and conducted at single centers. Due to the rarity of upper ureteral stricture cases, it is challenging for a single center to gather a sufficient number of samples. Therefore, it is crucial to conduct multicenter, large-sample studies with long-term follow-up to obtain more comprehensive data on the outcomes of UC. Additionally, comparative studies between minimally invasive surgeries and open surgeries are required to confirm the advantages, disadvantages, and long-term effects of both approaches. Ultimately, such studies will enable patients to make informed decisions and choose the most suitable method that will benefit them the most.

## Conclusion

UC is a safe, feasible, and effective surgical method for the treatment of complex upper ureteral stricture (including secondary renal pelvic junction obstruction) in adults or children. Robotic-assisted techniques are generally preferred for this procedure. However, prospective, multicenter and large-sample comparative studies with longer follow-up results are still needed.

## Data Availability

The original contributions presented in the study are included in the article/supplementary material. Further inquiries can be directed to the corresponding author.

## Declarations

### Research involving human and animal participants

This article does not contain any studies with human participants or animals performed by any of the authors.
